# Comprehensive assessment of the utilization of manure in China’s croplands based on national farmer survey data

**DOI:** 10.1038/s41597-023-02154-7

**Published:** 2023-04-19

**Authors:** Qingsong Zhang, Yiyan Chu, Yulong Yin, Hao Ying, Fusuo Zhang, Zhenling Cui

**Affiliations:** 1 College of Resources and Environmental Sciences; National Academy of Agriculture Green Development, China Agricultural University, 100193 Beijing, China; 2 Sanya Institute of China Agricultural University, 572025 Sanya, Hainan China

**Keywords:** Agriculture, Sustainability

## Abstract

China’s rapid increase in mass excreta and its environmental discharge have attracted substantial attention. However, cropland as a main destination of excreta utilization has not been extensively evaluated. Here, a national survey was used to assess the utilization of manure in croplands across China. The data included the inputs of manure nitrogen (N), phosphorus (P), and potassium (K) for cereals, fruits, vegetables, and other crops, along with the manure proportion of total N, P, and K inputs at the county level. The results showed that the manure N, P, and K inputs were 6.85, 2.14, and 4.65 million tons (Mt), respectively, constituting 19.0%, 25.5%, and 31.1% of the total N, P, and K, respectively. The spatial distribution of the manure proportion of total inputs was lower in Eastern China and higher in Western China. The results provide a detailed description of the utilization of manure nutrients in agricultural areas throughout China, which will serve as basic support for policymakers and researchers involved in future agricultural nutrient management in China.

## Background & Summary

Manure nutrient resources from human and livestock excretion have had a vital long-term role in ensuring food security and supporting the prosperity of human society, since before the appearance of chemical fertilizers^1,2^. Over the past few decades, the increasing global demand for meat and milk, particularly in developing countries, has promoted the rapid development of livestock production^3^, which has been accompanied by animal excretions that contain large amounts of nutrients^4–6^. However, the abundance of excrement has not been free of consequences; the overall excess of nitrogen (N) and phosphorus (P) in agricultural areas^2^ has led to massive nutrient discharges into the atmosphere and water bodies as environmental pollution^4,7,8^. Emerging economies (e.g., China and India) are becoming hotspots of nutrient overuse; their environments are experiencing significant impacts^9,10^. For example, animal numbers and chemical fertilizer use in 2012 were four-to-five-fold higher in China than in other counties^11^.

The number of smallholder farms in China has increased, and the high-input production of these farms continues to have a significant environmental impact^12^. On a global scale, China uses 9% of the world’s croplands to feed 18.3% of the world’s population, while consuming 25% of the world’s chemical N, P, and potassium (K) to ensure food security^3^. Before the 1980s, manure was the main input source for agricultural production in China; since then, chemical fertilizer inputs have greatly increased, whereas the proportion of manure input has sharply declined^5,13^. Additionally, because of its large population and continued economic growth, China has become the world’s largest livestock and poultry producer^5^. However, factors such as the gradual decoupling of livestock and crop production^6^, poor manure treatment technologies^14^, and an inefficient manure management chain^15^ have hindered the full utilization of the large amount of available manure. Specifically, only 17–38% of manure N, P, and K nutrients were returned to the cropland in China in 2010^15^.

Thus far, the efficiency of manure management has been limited by several key problems. First, it is difficult to clearly determine the amount of manure placed into cropland by Chinese farmers. Although some studies have estimated the amount and proportion of manure nutrients entering cropland based on models or regional surveys^5,16^, these estimates do not provide accurate descriptions because of crop cultivation diversity and the absence of primary data regarding farmers at the national level. Second, crop production in China has a specific spatial distribution; however, the lack of information regarding the spatial distribution of manure input to cropland leads to increased uncertainty when assessing nutrient management. Third, the proportions of manure and chemical fertilizer input affect crop yields, reactive N losses, and greenhouse gas emissions^17^, but there are few reports concerning the national spatial variability in fertilizer use in China. The management of these problems would be particularly beneficial for efforts to optimize China’s manure resources and protect the environment.

**Fig. 1:**
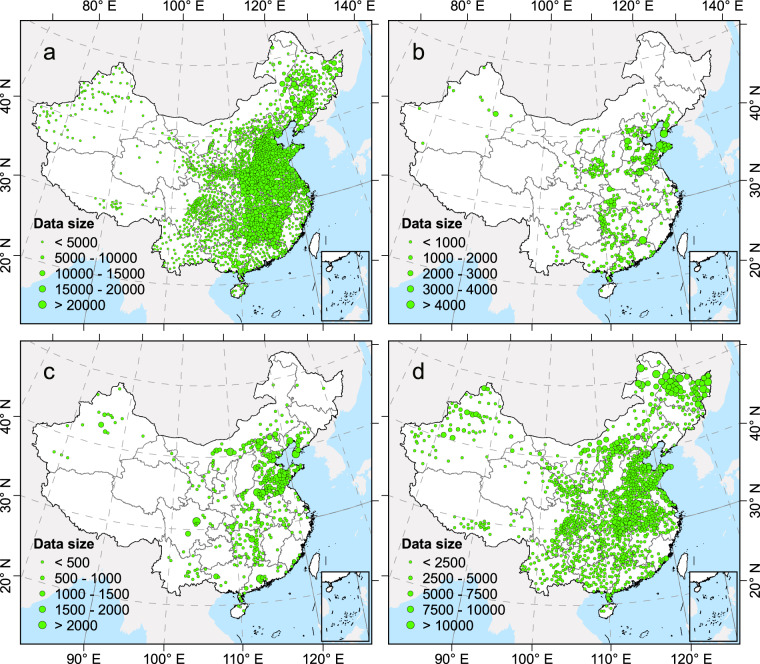
Distribution of national survey data at the county level in China from 2005 to 2014. (**a**–**d**) are cereals, fruits, vegetables, and other crops, respectively. Each point represents a county, and the size of the point represents the amount of data.

There are hundreds of millions of households engaged in agriculture in China^18^; thus, a large amount of farmer survey data is necessary to clearly understand the production practices of these farmers. In this study, national survey data of >6.6 million farmers were collected from 2005 to 2014; these farmers were involved in the production of 53 crops in major agricultural regions in China (Table 1). First, the farmers were divided into four groups according to crop type: cereals, fruits, vegetables, and others (Fig. 1); the total manure N, P, and K input to the cropland for each crop type was extensively quantified at the county level. Second, based on county-level statistical data, the manure inputs for the four crop types were calculated. Finally, in combination with chemical fertilizer input data from the national survey, the county-level spatial variation of the proportion of manure input was established. Our results provide a clear perspective for manure nutrient management in agricultural production, as well as detailed support for environmental impact assessment at the spatial level in China.

**Table 1:** The list of 53 crops in the national farmers survey in China.

Crop type	Crop name
Cereals	Maize; Rice; Wheat
Fruits	Apple; Banana; Orange; Citrus; Grape; Pear; Peach; Litchi; Muskmelon; Cherry; Strawberry; Watermelon
Vegetables	Cabbage; Carrot; Cucumber; Radish; Tomato; Pepper; Kidney bean; Ginger; Eggplant; Celery; Garlic
Others	Cotton; Chinese chestnut; Faba bean; Tea; Soybean; Barley; Potato; Sweet potato; Sugarcane; Sorghum; Millet; Flaxseed; Peanut; Mung bean; Cassava; Buckwheat; Highland barley; Mulberry; Sugar beet; Pea; Sunflower; Tobacco; Rapeseed; Hulless oat; Jujube; Sesame; Ramie

## Methods

### National farmers survey

To clarify the actual nutrient inputs of various crops in China, a national survey of 6.6 million farmers in 2,013 counties was conducted from 2005 to 2014. Farmers were interviewed face-to-face by local agricultural extension agents, using a standardized questionnaire that consisted of closed-ended questions. These questions were designed to obtain information regarding crop type, yields, manure source, and fertilizer use. The farmers surveyed for each crop were selected from villages and counties. Three to 10 villages were identified from each county, and 30–120 farmers were randomly selected from each village. In this survey, villages and farmers were randomly selected. Because a single farmer may plant more than one crop, 11.1 million data points were collected. In this study, 53 crops were categorized into cereals (3 crops: rice, maize, and wheat), fruits (12 crops), vegetables (11 crops), and others (27 crops)^19^. Information regarding chemical N, P, and K and manure application rates was used in this study.

### Assessment of nutrient input to cropland

In the farmers survey, manure input was recorded in forms such as pig slurry, cow slurry, farmyard manure, and compost. We used the nutrient concentration parameters from the Nutrient Content in Organic Fertilizers of China^20^ to calculate the N, P, and K nutrients in manure. Chemical nutrient inputs for specific crops in the survey compensated for the absence of valuable information regarding total fertilizer input. We calculated the initial total input of chemical fertilizer combined with the crop planting area at the county level. To ensure consistency with the total input of chemical fertilizer obtained from statistical data, the chemical fertilizer input per unit area for each crop in each county was corrected in the same manner used for the input of manure. To manage the missing nutrient input values of specific crops (or crop types) in specific counties, provincial or national (if province missing) mean values of the crop (or crop type) were used as substitute data. The total chemical N, P, and K inputs and crop planting area at the national and county levels were obtained from the Bureau and the 2012 yearbook statistics. The calculation of total manure and chemical N, P, and K input in each county were conducted as follows:

1 $$T{M}_{nutr,in}={\sum }_{1}^{m}(Ma{n}_{nutr,j}\times Are{a}_{j})$$

2 $$T{C}_{nutr,in}={\sum }_{1}^{m}(Che{m}_{nutr,j}\times Are{a}_{j})$$

where *TM*_*nutr, in*_ and *TC*_*nutr,in*_ are the total manure and chemical nutrient inputs of N, P, and K in each county, respectively; *Man*_*nutr,j*_ and *Chem*_*nutr,j*_ refer to the manure and chemical nutrient inputs per planting area of each crop (types), respectively; *j* (1, 2, 3… *m*) is the type of crop; and *Area*_*j*_ is the planting area of each crop (types).

The manure proportions of total nutrient input are estimated as follows:

3 $$Mpro{p}_{nutr,(j)}=T{M}_{nutr,in,(j)}/(T{M}_{nutr,in,(j)}+T{C}_{nutr,in,(j)})$$

where *Mprop*_*nutr,(j)*_ is the proportion of manure nutrients within total input (only considering chemical and manure input) for each county (or each crop); *TM*_*nutr,in,(j)*_ is the total manure nutrient input of N, P, and K for each county (or each crop); and *TC*_*nutr,in,(j)*_ is the total chemical nutrient input of N, P, and K in each county (or each crop).

### Data management

Data analysis was performed using Microsoft’s SQL server 2012 and Microsoft Office Excel 2019 (Microsoft Corp., Redmond, WA, USA). Correlations between crop planting area and number of farmers surveyed were determined via linear regression in IBM SPSS Statistics 25.0 (IBM Corp., Armonk, NY, USA). Vector layers of maps were acquired from the Resource and Environment Data Cloud Platform and National Catalogue Service for Geographic Information. All map-related operations were performed with ArcGIS 10.2 software.

## Data Records

**Fig. 2:**
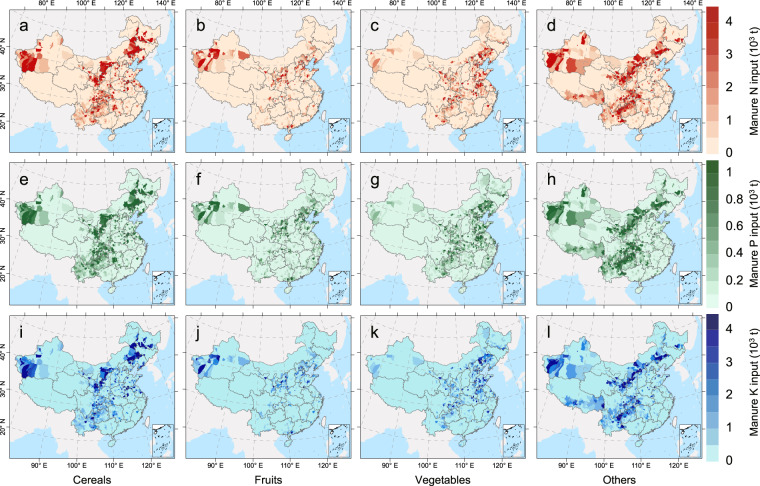
Spatial distribution of manure nutrient input for cereals, fruits, vegetables, and others for nitrogen (N) (**a**–**d**), phosphorus (P) (**e**–**h**), and potassium (K) (**i**–**l**), respectively, at the county level based on a national farmer survey conducted in China from 2005 to 2014.

**Fig. 3:**
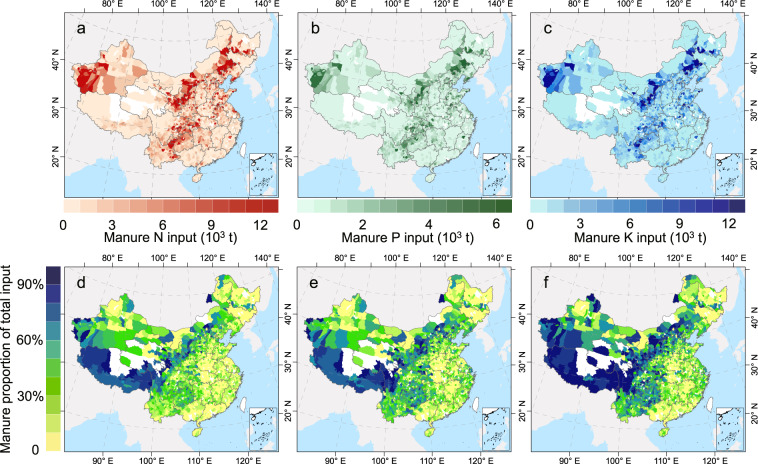
Total manure N (**a**), P (**b**), and K (**c**) input and manure proportion of the total N (**d**), P (**e**), and K (**f**) input at the county level in China from 2005 to 2014.

Our datasets were obtained in Excel file format with the following four sheets: “manure N”, “manure P”, “manure K”, and “manure percentage”. The first three sheets described the inputs (unit: tons) of manure N, P, and K into cropland (Fig. 2), respectively; the last sheet provided the manure proportions of total nutrient input (Fig. 3). These datasets contained the following fields: ProvCode, representing the provincial administrative division code; PAC2015, representing the 2015 county-level administrative division code, obtained from the Ministry of Civil Affairs of the People’s Republic of China; and “TM_Cereals”, “TM_Fruits”, “TM_Vegetables”, and “TM_Others” corresponding to the total manure N, P, and K inputs for the four crop types. In the sheet “manure percentage,” “Mprop_N”, “Mprop_P”, and “Mprop_K” were the manure nutrient proportions of total N, P, and K inputs, respectively. Blank fields indicated municipal districts and some counties without cropland. Here, we provide vector layers of China’s county-level administrative divisions, packaged in a compressed file called “ChinaCounty”; all results can be revealed by matching the shared PAC2015 field in the layers and Excel datasets. The data can be accessed from National Tibetan Plateau Data Center^21^ and processed in ArcGIS.

## Technical Validation

**Fig. 4:**
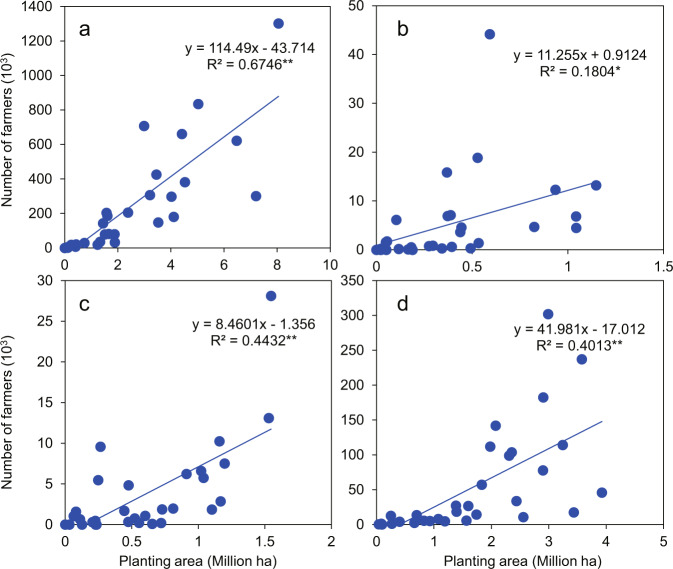
Correlation between crop planting area and number of farmers surveyed for (**a**) cereals, (**b**) fruits, (**c**) vegetables, and (**d**) others at the provincial level in China. *p < 0.05, **p < 0.01.

This study used a large data sample to evaluate the utilization of manure in China, with a particular focus on clarifying the spatial distribution of manure input in croplands. Uncertainty in the data is mainly related to the data reliability, as well as the coverage and representativeness of the nationwide survey. First, the nutrient application rates were determined by surveying millions of farmers throughout China in a project supported by the Chinese government; many universities and local agricultural extension agents participated in this work, along with numerous professional teachers and university students. The data were carefully screened and subjected to extensive quality control; they have been published in high-quality academic journals^19,22,23^. Second, the national survey covered the main provinces in which specific crops were planted. The four types of crops demonstrated a significantly positive correlation between the number of farmers surveyed and the crop planting area (Fig. 4). This relationship indicated that the spatial distribution of major crops was reflected in the national survey data. Third, when evaluating total nutrient inputs, for counties where nutrient inputs per hectare were missing, the mean value in the surrounding province was used to minimize uncertainty in the results.

## Data Availability

No specific code was used to produce the data described in this manuscript.
